# Efficient expression of fusion human epidermal growth factor in tobacco chloroplasts

**DOI:** 10.1186/s12896-022-00771-5

**Published:** 2023-01-07

**Authors:** Yunpeng Wang, Jieying Fan, Zhengyi Wei, Shaochen Xing

**Affiliations:** 1 Institute of Agricultural Biotechnology, Jilin Academy of Agricultural Sciences, Changchun, 130033 China; 2 Maize Research Institute, Guangxi Academy of Agricultural Sciences, Nanning, 530007 China

**Keywords:** EGF, Chloroplast transformation, Tobacco, GFP, Molecular farming

## Abstract

**Background:**

Chloroplast transformation is a robust technology for the expression of recombinant proteins. Various types of pharmaceutical proteins including growth factors have been reported in chloroplasts via chloroplast transformation approach at high expression levels. However, high expression of epidermal growth factor (EGF) in chloroplasts with the technology is still unavailable.

**Results:**

The present work explored the high-level expression of recombinant EGF, a protein widely applied in many clinical therapies, in tobacco chloroplasts. In this work, homoplastic transgenic plants expressing fusion protein GFP-EGF, which was composed of GFP and EGF via a linker, were generated. The expression of GFP-EGF was confirmed by the combination of green fluorescent observation and Western blotting. The achieved accumulation of the recombinant fusion GFP-EGF was 10.21 ± 0.27% of total soluble proteins (1.57 ± 0.05 g kg^− 1^ of fresh leaf). The chloroplast-derived GFP-EGF was capable of increasing the cell viability of the NSLC cell line A549 and enhancing the phosphorylation level of the EGF receptor in the A549 cells.

**Conclusion:**

The expression of recombinant EGF in tobacco chloroplasts via chloroplast transformation method was achieved at considerable accumulation level. The attempt gives a good example for the application of chloroplast transformation technology in recombinant pharmaceutical protein production.

**Supplementary Information:**

The online version contains supplementary material available at 10.1186/s12896-022-00771-5.

## Background

Epidermal growth factor (EGF), which has multiple biological activities, plays essential roles in various physiological and pathological processes in vertebrates [1, 2]. EGF can be utilized in the treatments of numerous kinds of wounds [3, 4]. Recombinant EGF (rEGF) is reported to be expressed in various of expression platforms including prokaryotic [5, 6, 7, 8] and eukaryotic[9, 10, 11, 12, 13, 14, 15, 16, 17, 18, 19, 20, 21, 22] systems. Plants, the large family of multicellular eukaryotes, are considered as efficient expression platforms for the expression of recombinant proteins [23, 24]. The plant expression platforms have many merits compared to other platforms, such as lower cost; easier scaling-up; easier storage; easier to find employees with professional skills and experiences in plant culture, harvesting and processing; sufficient post-translational modifications and less risks of biosecurity. There have been numerous reviews summarized in the field [25, 26, 27, 28, 29, 30, 31, 32, 33, 34, 35, 36, 37, 38, 39, 40, 41]. Many types of recombinant proteins, including industrial proteins (e.g., cellulase) [42, 43], biopolymers (e.g., spidroin) [44], and pharmaceutical proteins (e.g., EGF) [25] have been reported to be successfully expressed in plants.

The successful expression of EGF has been reported in tobacco (*Nicotiana tabacum* and *N. benthamiana*) [9, 10, 15, 19, 45, 46], tomato [12], soybean [16], potato [47], rice [20, 48], and saffron [21]. The strategies for the expression of EGF in plants were either transient or stable. The transient methods reported used either plant-virus-based vectors [9, 46] or mini-Ti vectors [15]. The stable expression of EGF in plants adopted both nuclear [9, 11, 12, 13, 16, 20, 21, 45, 46, 47, 48] and chloroplast [10, 19, 49] transformation. The expression levels varied from 0.001% to 7.8% of total soluble proteins (TSP). Chloroplasts transformation has been proven to be a powerful expression strategy for recombinant protein expression by numerous of advantages compared to nuclear transformation: (1) High expression level due to large numbers of transgenes; (2) Site-specific integration of exogenous genes via site-specific homologous recombination; (3) Multiple genes transformation at the same time; (4) Regulation of exogenous genes in polycistron strategy and (5) Maternal inheritance of transgenes in many plant species for avoiding the gene flow risks. Details on these advantages have been illustrated by Lössl and Waheed [50] and other reviews [51, 52, 53, 54]. Many proteins have been successfully expressed in plant plastids and summarized in previous reviews [41, 55, 56, 57, 58, 59, 60, 61, 62, 63, 64]. Up to date, there have been three reports that presented the expression of EGF in plant chloroplasts. Wirth et al. explored the expression status of EGF in tobacco chloroplasts, with the results suggesting that the expression of EGF could only be detectable when fused with the 186 aa of the N-terminal β-glucuronidase (GUS) [10]. Interestingly, the accumulation of EGF was higher in the dark than in the light. Nevertheless, the maximum of the expression obtained by this method was 0.1 ng g^− 1^ of fresh leaf. Morgenfeld et al. found it is beneficial to the accumulation of recombinant EGF in chloroplast when it was targeted to the thylakoid lumen by using a signal peptide (Str) [19]. However, the translocation from stroma to thylakoid lumen altered the solubility of EGF, and it was detectable only in insoluble fraction. In the recent report [49], Wang et al. achieved free rEGF in tobacco chloroplasts with the accumulation around 0.124% to 0.165% TSP of fresh biomass. Moreover, the native biological activity of rEGF from plant chloroplasts was demanstrated in this work.

As mentioned above, high level of EGF expression in chloroplast is not reported. In this paper, we reported the efficient expression of EGF in chloroplasts in a fusion status. In this work, the green fluorescent protein was adopted as a stabilizer with its C-terminal fusing to the N-terminal of EGF. The fusion showed a high expression level in tobacco chloroplasts and exhibited a similar activity accordant with its commercial counterpart.

## Results

### Vector construction

**Fig. 1:**
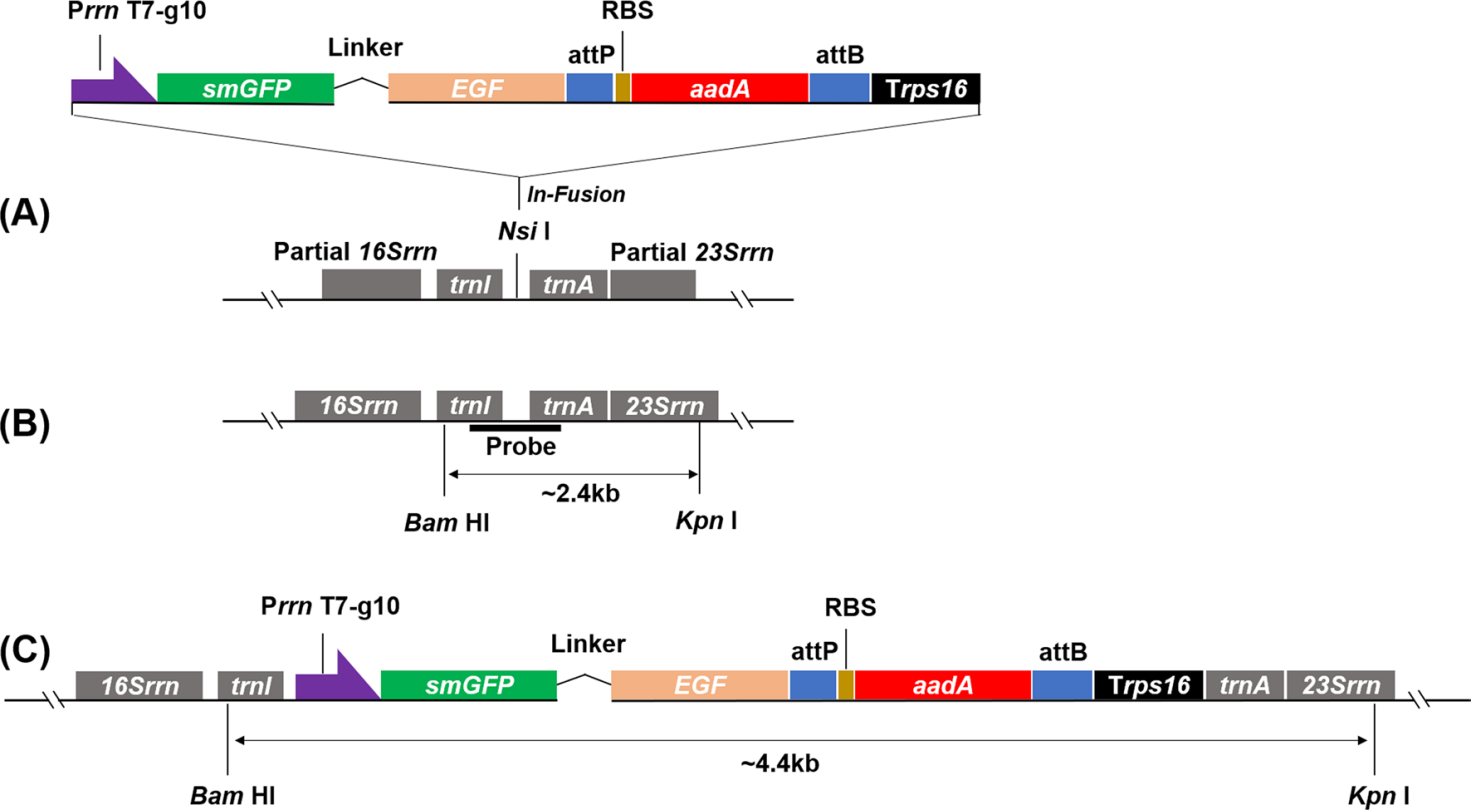
The structure construct pWYP21404 and the integration of tobacco chloroplast genome. **A** Expression cassette of the foreign genes. *smGFP*, the soluble, modified green fluorescent protein encoding sequence; linker, the linker peptide [(GSSSS)_3_-DDDDK] coding sequence; *EGF*, the epidermal growth factor encoding gene; *aadA*, the aminoglycoside adenyltransferase coding gene. P*rrn*, the promoter of the *rrn* opren of tobacco chloroplast genome; T7-g10, 5’ UTR of the T7 phage g10 gene; T*rps16*, the terminator of the tobacco plastid gene *rps16*. RBS, the ribosomal binding site of tobacco plastid *rbcL* gene; attP, attB, phage and bacterial recognize sites of the φ31 integrase. 16Srrn and 23Srrn, the ribosome 16 S rRNA and 23 S rRNA genes of tobacco chloroplast genome respectively; trnI and trnA, the transfer RNA of isoleucine and alanine encoding genes of tobacco chloroplast genome respectively. **B** The wild-type structure of flanking the target site (*Nsi* I) of the tobacco chloroplast. The restriction enzymes *BamH* I and *Kpn* I were used to digest the DNA for southern blotting analysis; the short, thick line indicates the probe used in DNA for southern blotting analysis. **C** The structure of the recombinant tobacco chloroplast genome

A tobacco chloroplast transformation vector pWYP21404 was constructed (Fig. 1A). The expression cassette P*rrn*-T7-g10-*smGFP*-*EGF*-attP-RBS-*aadA*-attB-T*rps16* was flanked by the sequences of *16S*-*trnI* and *trnA*-*23S* at its 3’ and 5’ terminal respectively. The fusion gene *smGFP-EGF* and selectable gene *aadA* were organized in the same cistron in the cassette. The genes *smGFP* and *EGF* were fused together with a linker [(GSSSS)_3_-DDDDK] coding sequence, which was designed for the cleavage of the two parts of the fusion protein in the future. The promoter P*rrn* was employed to launch transcription of the foreign genes and the terminator T*rps16* was adopted to stop the transcription. The 5’ UTR of the g10 gene of the T7 phage (T7-g10) was employed to enhance the expression of the fusion protein. The φ31 integrase recognize sites attP and attB were placed at the 3’ and 5’ terminals of *aadA* respectively for the future removal of the selectable gene from the recombinant chloroplast genome. An RBS sequence was placed between attP and *aadA* to help the translation of the *aadA* mRNA.

### Generation of transplastomic tobacco plants and the inheritance of foreign genes

**Fig. 2:**
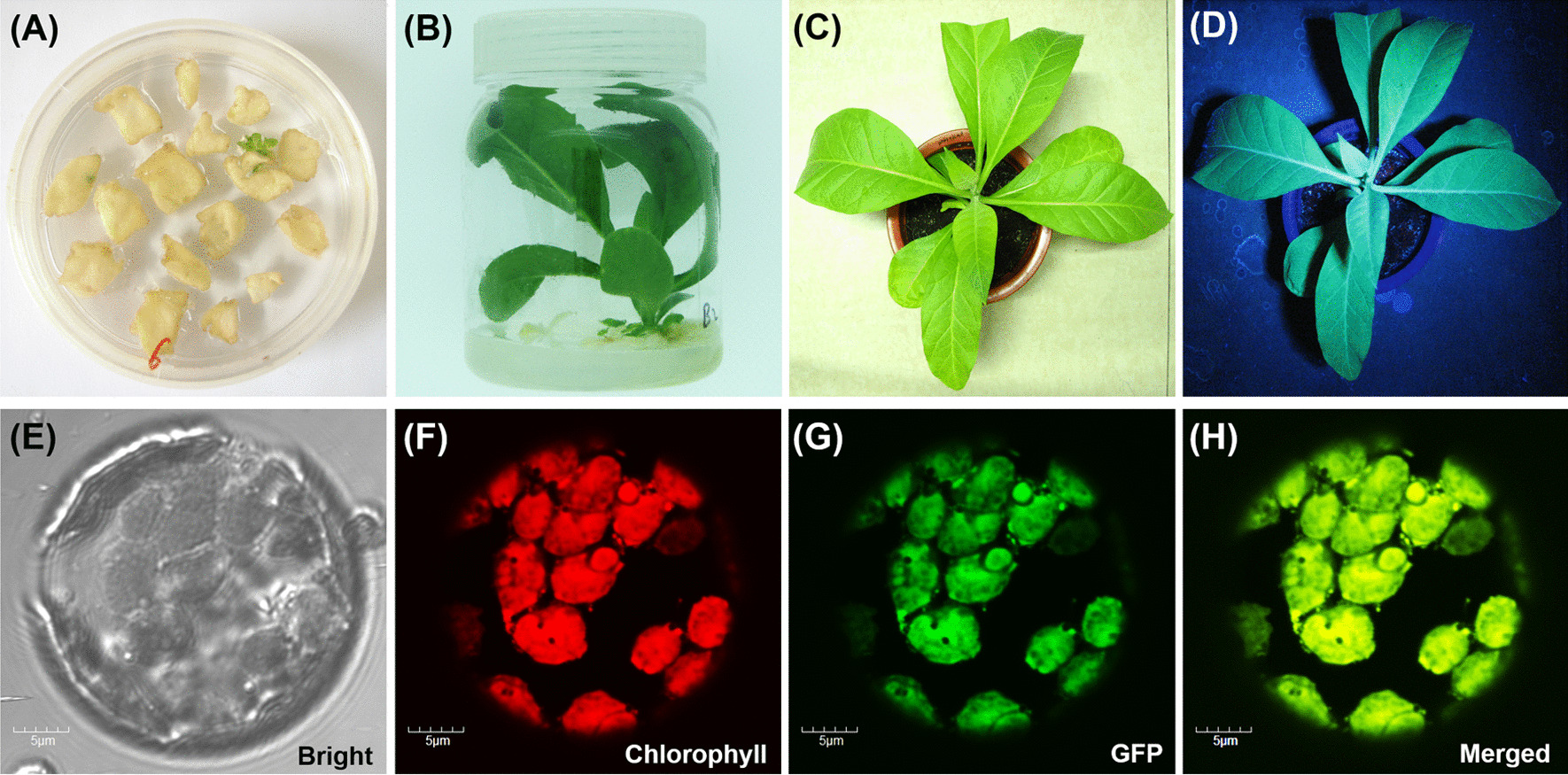
Generation of transplastomic tobacco and the expression of GFP in tobacco plants. **A** resistant shoot regenerated for explants on selectable medium. **B** A transplastomic plant grew on selectable medium. **C** A transplastomic plant in soil in natural light. **D** A transplastomic plant in UV light. **E–H** A transplastomic protoplast observed by laser confocal fluorescent microscope

**Fig. 3:**
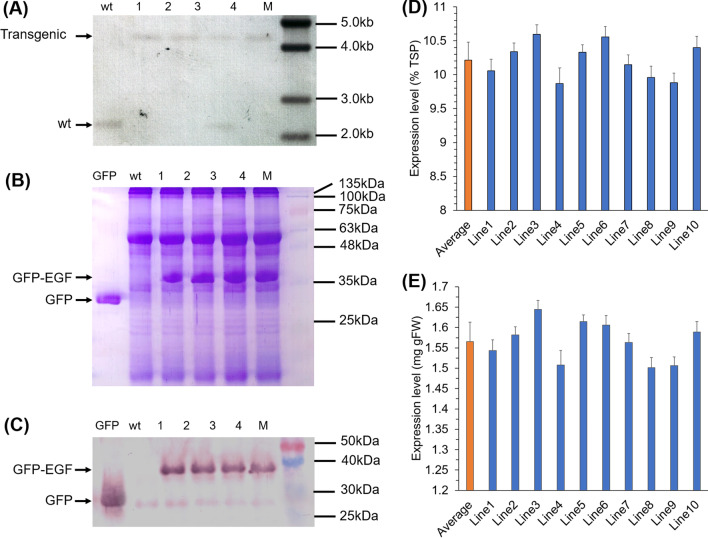
Southern blotting analysis and expression of GFP-EGF in tobacco plants. **A** Southern blotting used the probe indicated in Fig. 1, five micrograms of DNAs for each were digested by *Bam* HI/*Kpn* I and used. GFP, the GEP standard protein; wt, wild-type plant; 1–4, transplastomic plants. **B, C** The SDS-PAGE and the Western blotting analysis of the GFP-EGF expression. Wt, wild-type plant; 1–4, transplastomic plants. **D, E** The Expression level of GFP-EGF in transplastomic tobacco plants. Six tests were repeated for each line, and the data were shown as mean ± SD. Average, the average expression of the 10 tested lines; line1-line10, different transplastomic tobacco lines

Spectinomycin resistant tobacco shoots were regenerated from the bombarded leaf pieces on the selectable medium with spectinomycin supplemented (Fig. 2A, B). The emission of green fluorescence from the transplastomic plant under UV light indicated the successful integration of the heterologous genes (Fig. 2D), and wild type phenotype was observed under normal light (Fig. 2C). The emission of green fluorescence from transplastomic protoplasts was also observed (Fig. 2E–H). The T1 generation seedlings grew normally on media either with or without spectinomycin and emitted green fluorescence upon the exposure with UV light (Additional file 1: Fig. S2). Whereas, the wild-type seedlings were bleached on the spectinomycin-containing medium and emitted red fluorescence of chlorophyll in UV light on medium without spectinomycin supplemented (Additional file 1: Fig. S2). These results suggested the inheritance of the foreign genes from transgenic plants to their progenies. The results from Southern blotting showed that a recombinant specific band of approximately 4.1 kb in transplastomic plants was detected but an approximately 2.4 kb-band was found in the wild-type plant (Fig. 1B, C), which supported the concept that the detected transplastomic plants #1, 2 and 4, were homoplasmic (Fig. 3A).

### Expression of GFP-EGF

The expression of the fusion protein was determined by green fluorescence observation with the combination of SDS-PAGE analysis and Western blotting analysis. The transgenic plants showed green fluorescence emission both *in planta* and in vitro (Fig. 2D, Additional file 1: Figs. S1, S2), which provided strong evidences for the expression of GFP-EGF fusion. The coomassie brilliant blue stained SDS-PAGE gel showed an additional protein band with large amount protein with a molecular weight of about 35.6 kDa, which fit to the expected molecular weight of GFP-FGF. (Fig. 3B). The result from Western blotting analysis also showed a specific band of the same molecular weight (Fig. 3C). The expression of GFP-EGF was further quantified by ELISA. The average expression level was 10.21 ± 0.27% TSP, namely, an average expression of 1.57 ± 0.05 g kg^− 1^ of fresh leaf (Fig. 3D, E).

### Biological activity of GFP-EGF

**Fig. 4:**
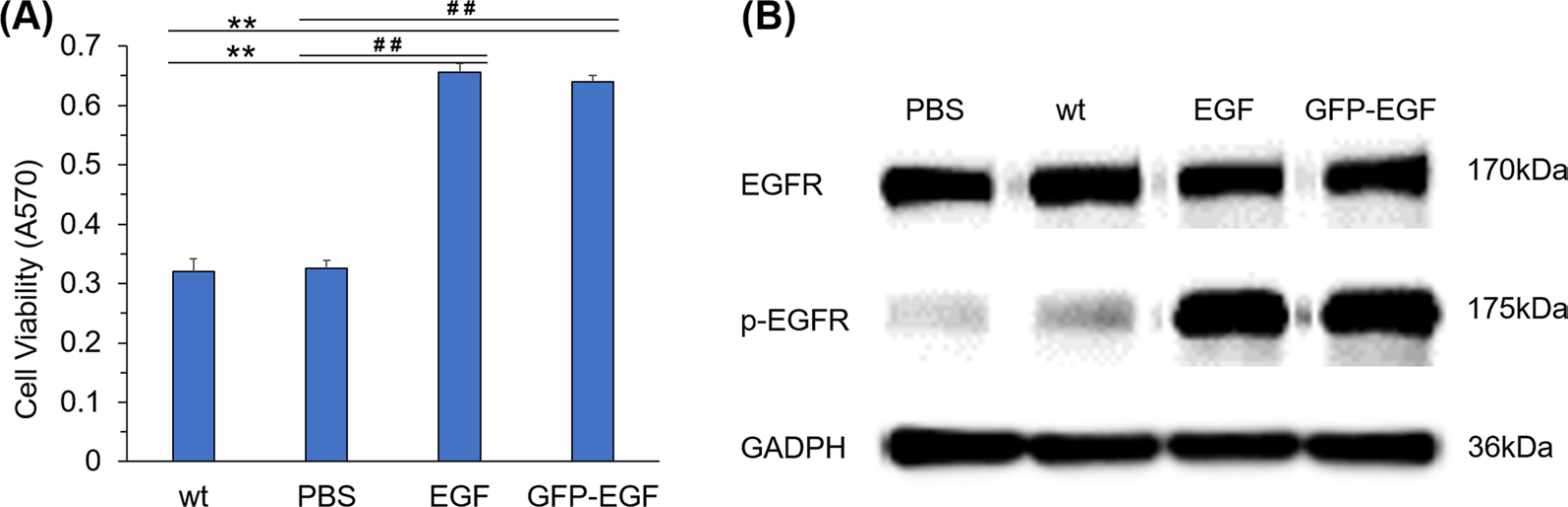
Biological activity of GFP-EGF. Cell viability (**A)** and the phosphorylation of EGFR (**B**) in A549 cells treated by GFP-EGF. Wt, treatments with TSP of wild-type plant; PBS, the blank treatments; EGF, treatments of standard EGF; GFP-EGF treatments with TSP of transplastomic plant. **, ##, *p* < 0.05, compared to wt and PBS respectively

The cell viability test was performed on NSLC cell line A549 to primarily assess the bioactivity of the fusion protein GFP-EGF. The cell viability was much higher in the GFP-EGF treatments compared with the blank controls and the wild-type TSP controls, which was similar to the standard product (Fig. 4A). These data suggested that the GFP-EGF derived from tobacco chloroplasts was capable of conducting the biological activity of promoting the proliferation of cancer cells. Further investigation suggested that the treatment of GFP-EGF enhanced the phosphorylation level of EGFR in A549 cells (Fig. 4B), which subsequently promoted the cell proliferation. The combined data showed that GFP-EGF could play the role of natural EGF in cancer cells by mediating the phosphorylation of EGFR.

## Discussion

Although the expression of EGF has been reported in various plant species, the expression level remains different and unsteady among the data available. The first attempt of expression EGF in tobacco achieved only a maximum expression level of 0.001% TSP [45], and the efforts subsequently differed from the expression strategies. In tobacco, there were several reports showed different expression levels of EGF [9, 10, 11, 13, 14, 18, 19, 20, 45, 49], and highest expression level achieved was 6.24% TSP [15]. The accumulation of EGF in transgenic tomato was reported to be 3.78 ng g^−1^ in fruit [12] and 6.7 ± 3.1 to 129.0 ± 36.7 µg g-1 of dry weight (DW) in soybean seed [16]. In transgenic rice, EGF could occupy 1.8% TSP in normal cells, and in hypoxic transgenic seedlings, 7.8% TSP could be achieved [48]. Here, our data showed that 10.21 ± 0.27% TSP of GFP-EGF fusion protein was achieved in tobacco chloroplast, which is much higher than the previous reports.

Although chloroplast transformation strategy has been reported for the expression of EGF [10, 19, 49], the merit of the technology, i.e., high expression of recombinant protein, was not embodied in these efforts. EGF is an acidic protein with an isoelectric point of 4.6 which is stable in acidic environment. However, the chloroplast stroma is alkaline (in the light) or neutral (in the dark), which might be caused by the instability of EGF that affected the accumulation of EGF in chloroplasts stroma due to its natural properties. Therefore, the accumulation of EGF in the dark was higher than that in the light [10], same situation happened in the higher accumulation of EGF in the chloroplast thylakoid lumen [19], and although the accumlation of free rEGF in our previous work[73] was relatively higher, it was still insufficient for commercialization. Actually, the accumulation of EGF was higher in the subcellular locations with lower pH environments as mentioned above, e.g., the vacuole [15]. Thus, other strategies should be adopted to address the instability of EGF in chloroplast stroma. The status of EGF in expression host cells also affected the accumulation level. Several attempts have been examined to improve EGF accumulation. The data reported showed that, the free EGF in plant cells often achieved lower accumulation, but the GUS [10], Zera [20], cMyc-tag [48], or chloroplast thylakoid lumen target transit peptide [19] fusion helped to improve the accumulation. In this study, the GFP was adopted to stabilize EGF in chloroplast stroma due to its excellent stability in many occasions [65]. By combining with codon optimization, the expression level presented here was up to 10.21 ± 0.27% TSP (1.57 ± 0.05 g kg^− 1^ of fresh leaf), which gave a good example for the promising application of chloroplast transformation for rEGF production.

## Conclusion

The high expression of rEGF with biological activity in tobacco chloroplasts via chloroplast transformation method was achieved, and the expression level was demonstrated to be 10.21 ± 0.27% TSP. These give a good example for the application of chloroplast transformation technology in recombinant pharmaceutical protein production.

### Methods

#### Plant material and cultivation

Germination of tobacco (*Nicotiana tabacum cv.* Petite Havana) seeds and seedling culture was performed as described previously [66]. Leaves of two-week-old seedlings were collected for chloroplast transformation.

### Vector construction

An expression cassette was designed and artificially synthesized (Fig. 1A). The epidermal growth factor (EGF, NCBI accession No.: M26695) encoding gene (*EGF*) was codon-optimized accordant with the codon usage bias of tobacco chloroplast and fused to the 3’ end of GFP encoding gene (*smGFP*, NCBI accession No.: U70495) by a linker [(GSSSS)_3_-DDDDK] coding sequence. The fusion gene *GFP-EGF* was placed downstream of the promoter of *rrn* operon of tobacco chloroplast genome (P*rrn*) and the 5’ UTR of the g10 gene of the T7 phage (T7-g10) [67, 68], which was used to enhance the expression level. The selectable marker gene, *aadA*, was placed at the 3’ end of the fusion gene *smGFP-EGF* followed by a ribosomal binding site (RBS) of the *rbcL* gene from tobacco chloroplast genome. A φ31 integrase recognition site, attP, was inserted between the gene *smGFP-EGF* and the RBS, which was designed for the later removal of *aadA* gene from the recombinant chloroplast genome by combining the other integrase recognize site, attB. The attB sequence was at the 3’ end of the *aadA* gene and followed a terminator T*rps16*, the terminator of tobacco chloroplast *rps16* gene. The cassette was amplified by PCR method with high-fidelity DNA polymerase and inserted into the *Nsi* I site between the genes of *trnI* and *trnA* in the *16 S-trnI-trnA-23 S* fragment from the tobacco chloroplast genome [69] with an In-Fusion® HD Cloning Kit (ClonTech Laboratories, Inc., USA) to form the expression vector pWYP21404.

### Chloroplast transformation and generation of transplastomic tobacco plants

Young leaves of tobacco seedlings were used as explants for chloroplast transformation. The chloroplast transformation, plant regeneration, and homoplasmic selection were conducted according to the previous protocols [66]. The homoplasmic selection was performed for 3 to 4 rounds until the homoplasy was achieved. The homoplasmic lines were picked out by green fluorescence observation combined with Southern blotting analysis. The homoplasmic lines were transferred to soil and cultured in a greenhouse, seeds were collected for further investigations.

### Identification of homoplasmic transplastomic tobacco lines

The regenerated spectinomycin resistant plantlets were placed under the 365 nm UV light to observe the emission of green fluorescence. The lines with strong green fluorescent emission from 3- or 4-round selection were selected for further Southern blotting analysis. The total DNAs from transplastomic and wild-type plants were extracted for the Southern blotting analysis. The DNA samples (5 µg for each) were digested by restriction enzymes *Bam* HI and *Kpn* I. The DNA electrophoresis, transference to membrane, hybridization, and signal detection were carried out following the previously described protocols [66], using a DIG High Prime DNA Labeling and Detection Starter Kit I (Roche, Switzerland). A 988 bp of DNA fragment flanking the genes *trnI* and *trnA* (Fig. 1B) was adopted as hybridization probe. The probe was amplified from tobacco chloroplast genome by using the primers NtprobF (5’- AATGGAGCACCTAACAACGCATCTTC -3’) and NtprobR (5’- TAATGCGTTCCACTTATTGAACA GGG -3’).

### Expression analysis of GFP-EGF in tobacco chloroplasts

T1 seeds were germinated as described above and cultured on media with or without spectinomycin supplemented for observation of the inheritance of the foreign genes. The green fluorescence emission observation was performed for the visible investigation of the expression of the fusion protein GFP-EGF in transplastomic plants. The transgenic plants (seedlings) were exposed under UV light and a digital camera was adopted to take the pictures of fluorescents. Protoplasts were also isolated from transgenic plants for the in vitro green fluorescence observation by laser confocal fluorescence microscope methods followed the procedures reported previously [70].

TSPs were extracted from both transplastomic and wild-type plants followed the previous protocol described [71] for the further expression analyses of the GFP-EGF fusion. Fifteen micrograms of TSPs for each were used to carry out the SDS-PAGE analysis with a 12% separate gel and, the gel was later stained with Coomassie brilliant blue R250. Western blotting was conducted according to the procedure described previously [66] by using the monoclonal antibody against GFP (rabbit derived) and the horse radish peroxidase-labeled anti-rabbit IgG antibody (goat derived). The expression level was quantified by the ELISA method conducted in the sandwich way with a pair of polyclonal and monoclonal antibodies against GFP. The above analyses adopted a standard GFP as positive controls and wild-type TSPs as negative controls.

### Activity assessment of GFP-EGF

The non-small cell lung cancer (NSCLC) cell line A549 (purchased for National Collection of Authenticated Cell Cultures, Chinese Academy of Sciences, Shanghai) was adopted to assess the biological activity of the fusion protein GFP-EGF. Cell culture was carried out as described by Zhang et al. [72].

The cell viability test was firstly performed. Briefly, A549 cells (1 × 10^5^ ml^− 1^) were cultured in 96-well plates with 100 µL medium per well. The media contained 25 nmol l^− 1^ of EGF standard, TSP form transplastomic tobacco (containing 25 nmol l^− 1^ of GFP-EGF), TSP from wild-type tobacco (the same amount to the transgenic) or without supplement. The volumes were adjusted to 150 µl by using PBS. Cells were cultured for 24 h and detected with the thiazolyl blue tetrazolium bromide (MTT) method. Twelve wells were repeated for each treatment.

Phosphorylation of EGF receptor (EGFR) was further tested. The treatments were performed as followed: (1), the A549 cells with a density of 5 × 10^5^ ml^− 1^ were cultured in petri dishes (φ = 10 cm) for 14 h; (2), the medium was then replaced by fresh medium containing 25 nmol l^− 1^ of EGF standard or GFP-EGF (in the form of TSP mixture); (3), the cells were cultured for more 24 h. At the end of treatments, the TSPs of the cells were extracted and Western blotting was performed following the protocols described previously [73] to test the phosphorylation level of EGFR in cells. The PBS buffer was adopted the blank control. The antibodies against EGFR and phosphorylated EGFR (p-EGFR) were used. GADPH was employed as the internal reference.

## Supplementary Information

**Additional file 1.** Supplementary Materials.

**Additional file 2.** **Table S1.** Cell viability. **Table S2.** Expression of GFP-EGF.

**Additional file 3.** Raw Data.

## Data Availability

All data generated or analyzed during this study were included in this published article and its supplementary information files.
