# Supplementary material for: Efficient expression of fusion human epidermal growth factor in tobacco chloroplasts
Source: BMC Biotechnol. 2023 Jan 7;23:1. doi: 10.1186/s12896-022-00771-5 (PMC9824920; doi:10.1186/s12896-022-00771-5)
Supplement: Supplementary file 1 — Additional file 1. Supplementary Materials. [file 12896_2022_771_MOESM1_ESM.docx]

**Supplementary Materials**

**
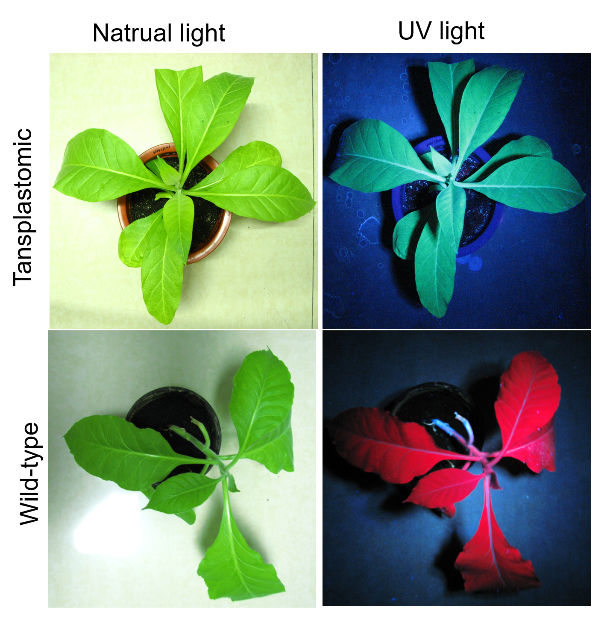
**

**Fig. S1.** Fluorescent observation of tobacco plants grew in soil. The plants were photographed three weeks later after transferring to soil in the greenhouse (natural light) or a dark room (UV light).

**
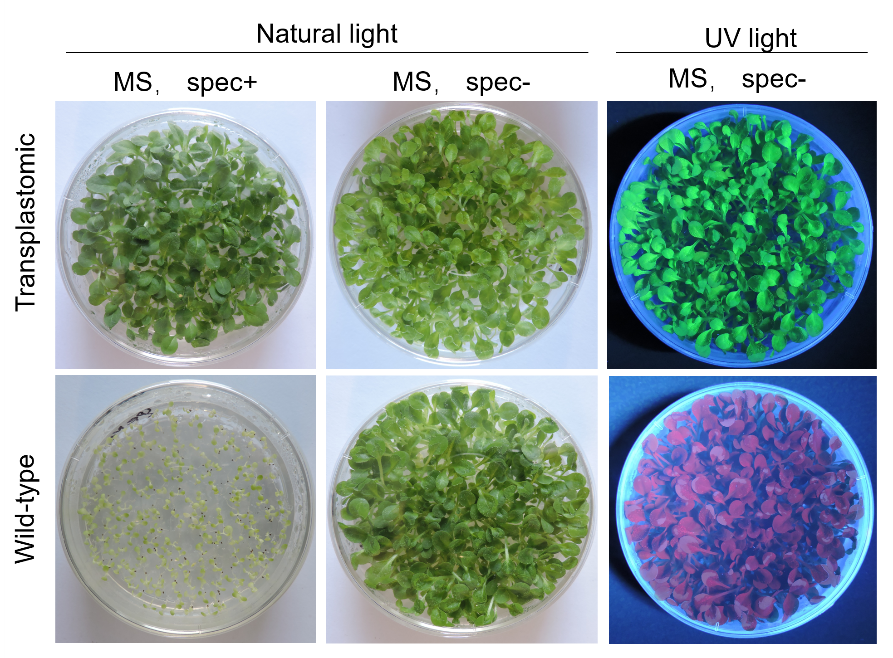
**

**Fig. S2.** Inheritance of foreign genes in the transplastomic plants. The plantlets were photographed three weeks later after germination on MS medium with (spec+) or without (spec-) spectinomycin in the culture room (natural light) or a dark room (UV light).
