# Supplementary material for: Efficient expression of fusion human epidermal growth factor in tobacco chloroplasts
Source: BMC Biotechnol. 2023 Jan 7;23:1. doi: 10.1186/s12896-022-00771-5 (PMC9824920; doi:10.1186/s12896-022-00771-5)
Supplement: Supplementary file 2 — Additional file 2. Table S1. Cell viability. Table S2. Expression of GFP-EGF. [file 12896_2022_771_MOESM2_ESM.docx]

**Table S1** Cell viability

| Well # | Samples (OD_570_) | | | | |
| --- | --- | --- | --- | --- | --- |
|  | Blank | PBS | wt | EGF | GFP-EGF |
| 1 | 0.011 | 0.323 | 0.331 | 0.664 | 0.632 |
| 2 | 0.008 | 0.312 | 0.312 | 0.678 | 0.657 |
| 3 | 0.002 | 0.345 | 0.351 | 0.653 | 0.641 |
| 4 | 0.005 | 0.356 | 0.319 | 0.637 | 0.653 |
| 5 | 0.008 | 0.331 | 0.27 | 0.628 | 0.637 |
| 6 | 0.002 | 0.321 | 0.324 | 0.671 | 0.648 |
| 7 | 0.004 | 0.327 | 0.322 | 0.656 | 0.627 |
| 8 | 0.003 | 0.318 | 0.335 | 0.647 | 0.642 |
| 9 | 0.003 | 0.327 | 0.34 | 0.649 | 0.631 |
| 10 | 0.001 | 0.31 | 0.326 | 0.662 | 0.626 |
| 11 | 0.009 | 0.324 | 0.31 | 0.651 | 0.649 |
| 12 | 0.002 | 0.314 | 0.308 | 0.674 | 0.639 |
| mean | 0.004833 | 0.325667 | 0.320667 | 0.655833 | 0.640167 |
| SD | 0.003326 | 0.013432 | 0.020375 | 0.014899 | 0.010125 |

The OD_570_ data of the MTT assay for the cell viability treated by EGFs. Blank, blank wells without cells on the 96-well microplate; PBS, the blank treatments; wt, treatments with TSP of wild-type plant; EGF, treatments of standard EGF; GFP-EGF treatments with TSP of transplastomic tobacco plant.

**Table S2** Expression of GFP-EGF

|  | GFP-EGF content (%) | | | | | | | | GFP-EGF content (mg/gFW) | |
| --- | --- | --- | --- | --- | --- | --- | --- | --- | --- | --- |
| Sample | Repeat A | Repeat B | Repeat C | Repeat D | Repeat E | Repeat F | Mean | SD | SD | Mean |
| Line1 | 10.04 | 10.08 | 9.82 | 10.22 | 10.26 | 9.91 | 10.055 | 0.171085 | 0.026259 | 1.543274917 |
| Line2 | 10.37 | 10.31 | 10.25 | 10.19 | 10.33 | 10.57 | 10.33667 | 0.130639 | 0.019994 | 1.582026833 |
| Line3 | 10.41 | 10.74 | 10.56 | 10.47 | 10.61 | 10.77 | 10.59333 | 0.143481 | 0.022271 | 1.644261889 |
| Line4 | 10.03 | 9.84 | 9.94 | 10.18 | 9.57 | 9.66 | 9.87 | 0.228736 | 0.034951 | 1.508136 |
| Line5 | 10.4 | 10.32 | 10.13 | 10.38 | 10.43 | 10.33 | 10.33167 | 0.107223 | 0.016754 | 1.614322917 |
| Line6 | 10.81 | 10.45 | 10.37 | 10.63 | 10.48 | 10.59 | 10.555 | 0.156684 | 0.02384 | 1.60594325 |
| Line7 | 10.18 | 10.31 | 10.13 | 9.91 | 10.27 | 10.09 | 10.14833 | 0.143167 | 0.02205 | 1.563012472 |
| Line8 | 9.93 | 10.21 | 9.82 | 10.07 | 9.97 | 9.76 | 9.96 | 0.164438 | 0.024786 | 1.501304 |
| Line9 | 9.96 | 10.02 | 9.89 | 9.68 | 9.73 | 10 | 9.88 | 0.143527 | 0.021878 | 1.506041333 |
| Line10 | 10.67 | 10.42 | 10.31 | 10.47 | 10.18 | 10.34 | 10.39833 | 0.166303 | 0.025417 | 1.589211944 |
| Mean |  |  |  |  |  |  | 10.21283 |  |  | 1.565593307 |
| SD |  |  |  |  |  |  | 0.268128 |  |  | 0.047439195 |

Ten homoplastic transplastomic plants was harvested to test the expression of GFP-EGF. ELISA in the sandwich way with a pair of polyclonal and monoclonal antibodies against GFP was adopted for the testing and six technical repeats were carried out.
