# Supplementary material for: Efficient expression of fusion human epidermal growth factor in tobacco chloroplasts
Source: BMC Biotechnol. 2023 Jan 7;23:1. doi: 10.1186/s12896-022-00771-5 (PMC9824920; doi:10.1186/s12896-022-00771-5)
Supplement: Supplementary file 3 — Additional file 3. Raw Data. [file 12896_2022_771_MOESM3_ESM.docx]

**Raw Data**


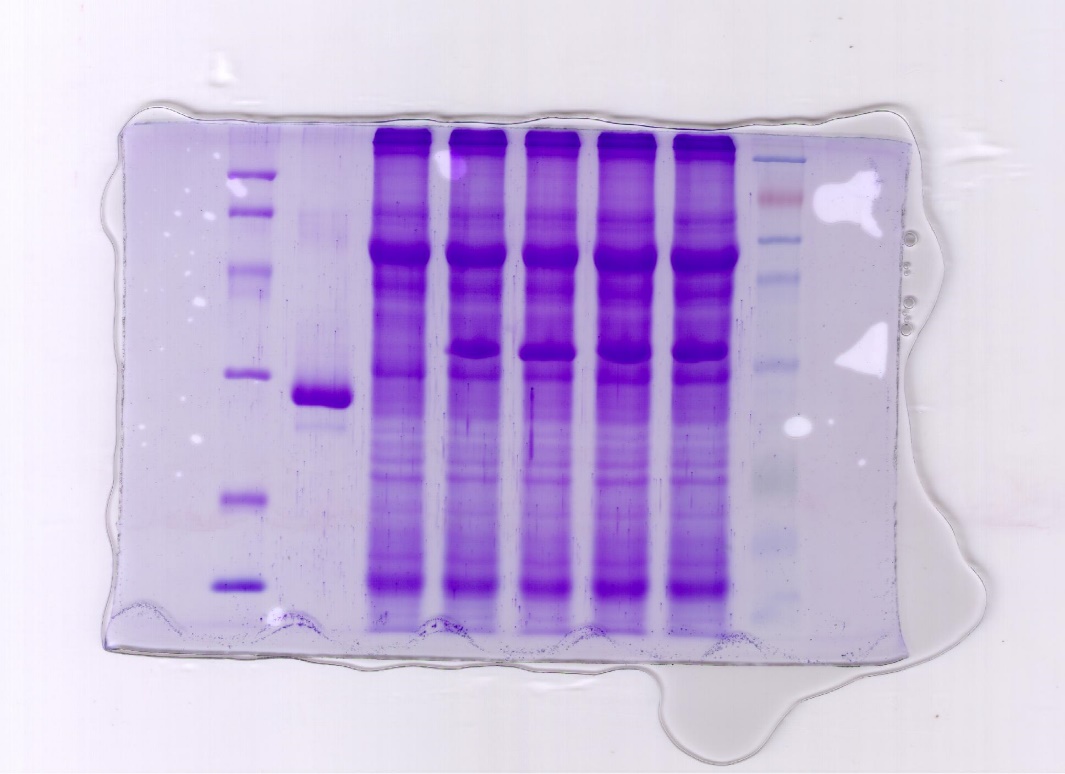


**Fig. RD1** GFP-EGF SDS-PAGE gel

Fifteen micrograms of TSPs for each were used to carry out the SDS-PAGE analysis with a 12% separate gel and, the gel was later stained with Coomassie brilliant blue R250. The picture was shot by a digital camera.


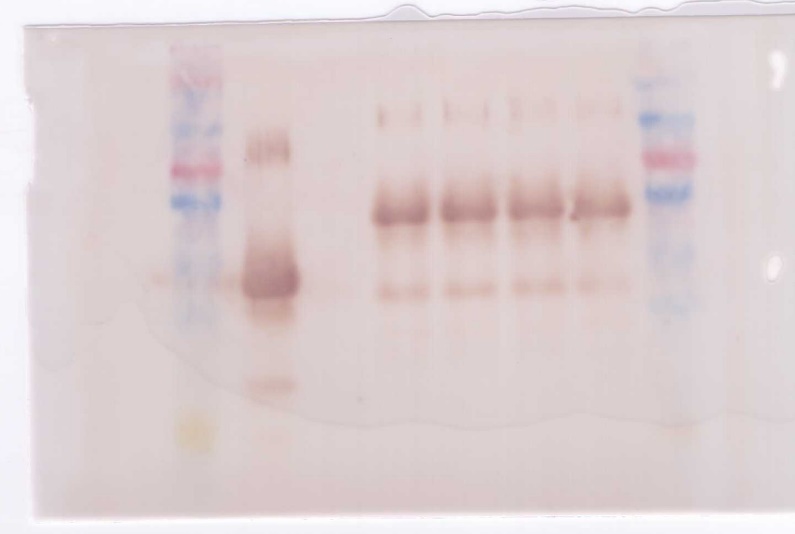


**Fig. RD2** GFP-EGF Western blotting image

Fifteen micrograms of TSPs per sample was used for Western blotting and the monoclonal antibody against GFP (rabbit derived) and the horse radish peroxidase-labeled anti-rabbit IgG antibody (goat derived) was used.


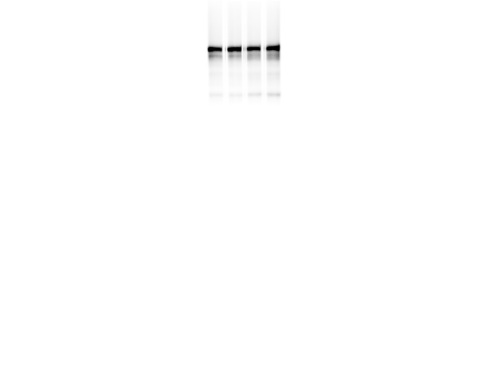

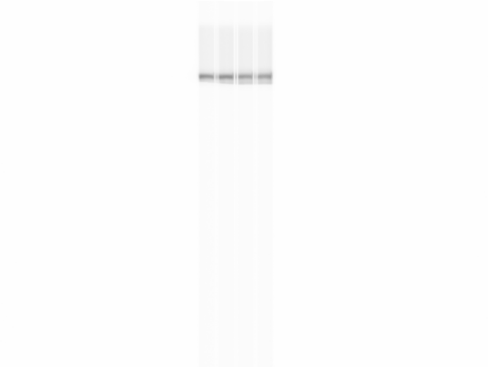


170kDa——

170kDa——


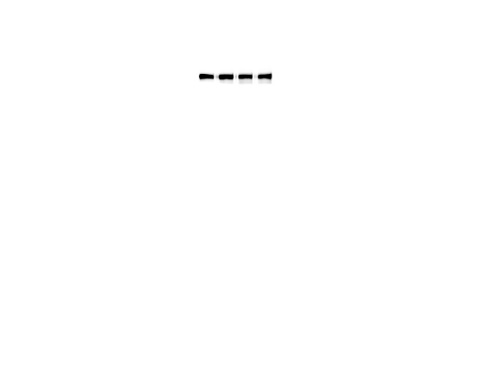

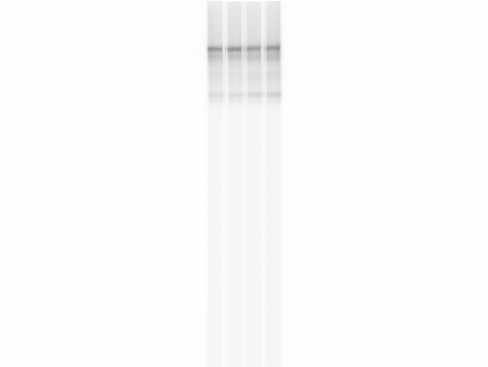


170kDa——

170kDa——

**Fig. RD3** EGFR Western blotting images

The western images with different exposures for the amount of EGFR testing in the treated NSCLC cell line A549.


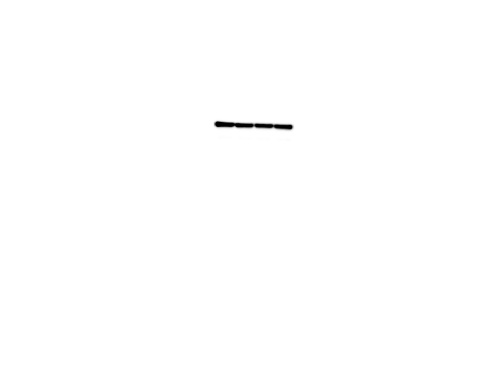

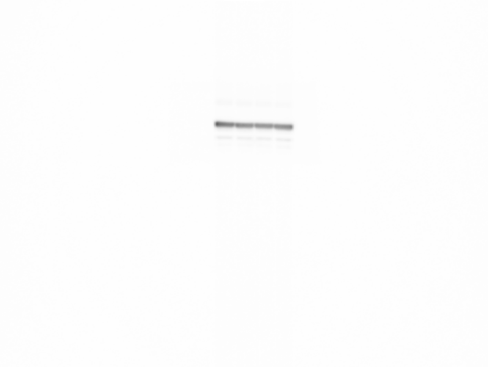


36kDa——

36kDa——

**Fig. RD4** GADPH Western blotting images

The western images with different exposures for the amount of GADPH testing in the treated NSCLC cell line A549.


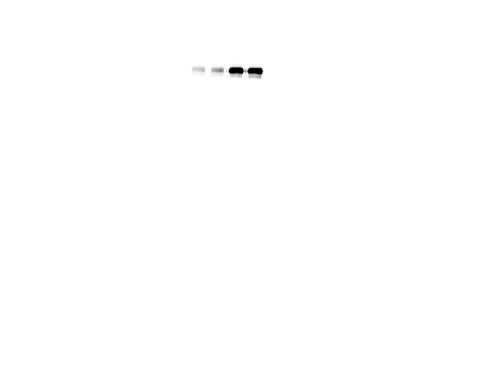

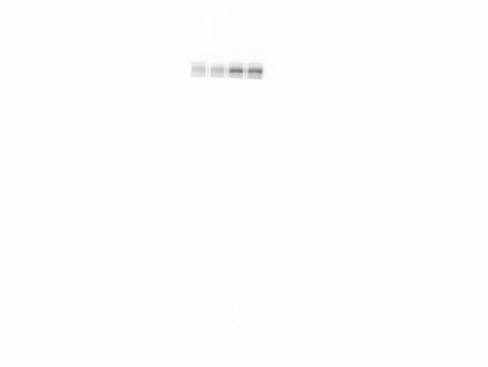


~175kDa——

~175kDa——


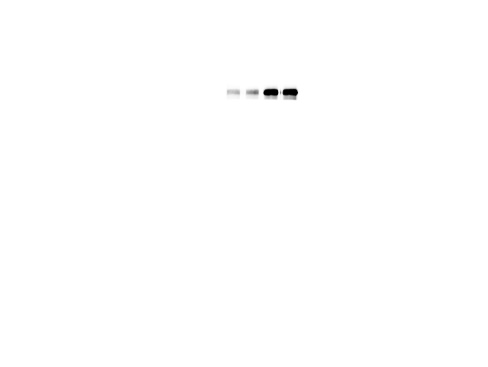

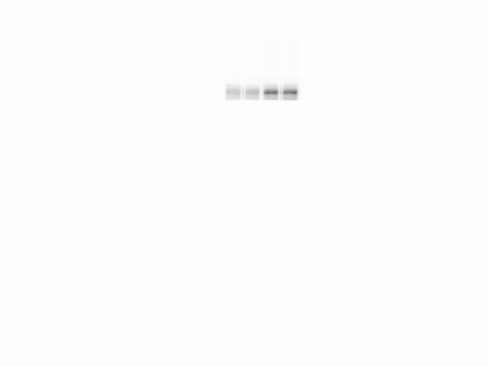


~175kDa——

~175kDa——


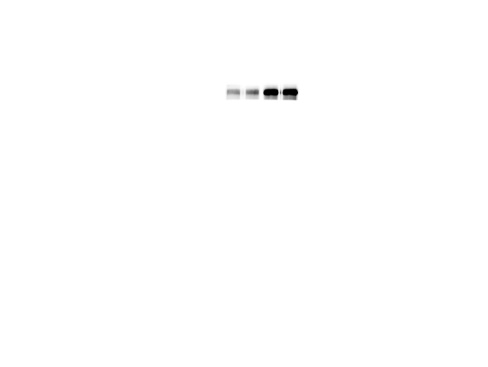

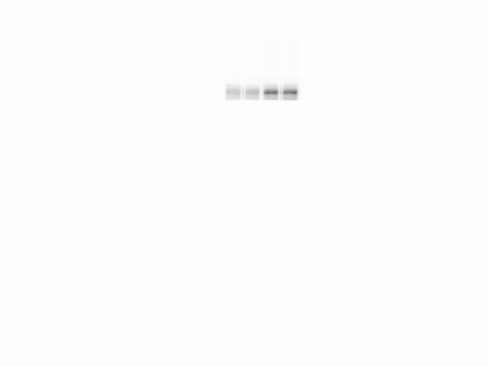


~175kDa——

~175kDa——

**Fig. RD5** phosphorylated EGFR (p-EGFR) Western blotting images

The western images with different exposures for the amount of p-EGFR testing in the treated NSCLC cell line A549.
